# Production and Clinical Evaluation of Norwalk GI.1 Virus Lot 001-09NV in Norovirus Vaccine Development

**DOI:** 10.1093/infdis/jiz540

**Published:** 2019-10-20

**Authors:** Roberto Mateo, Lisa C Lindesmith, Shaily J Garg, Keith Gottlieb, Karen Lin, Sara Said, Juan S Leon, Amy C Sims, David J Weber, Ralph S Baric, Sean N Tucker, David N Taylor

**Affiliations:** 1 Vaxart, Inc., South San Francisco, California, USA; 2 Department of Epidemiology, University of North Carolina, Chapel Hill, North Carolina, USA; 3 Hubert Department of Global Health, Rollins School of Public Health, Emory University, Atlanta, Georgia, USA; 4 Division of Infectious Diseases, University of North Carolina, Chapel Hill, North Carolina, USA

**Keywords:** antibody-secreting cells, blockade antibody, controlled human infection model, human challenge study, human norovirus

## Abstract

**Background:**

Human noroviruses (HuNoV) are the leading cause of gastroenteritis. No vaccine is currently available to prevent norovirus illness or infection. Safe, infectious challenge strains are needed to assess vaccine efficacy in the controlled human infection model (CHIM).

**Methods:**

A stock of HuNoV strain Norwalk virus ([NV] GI.1) was prepared. Healthy, genetically susceptible adults were inoculated with NV Lot 001-09NV and monitored for infection, gastroenteritis symptoms, and immune responses.

**Results:**

Lot 001-09NV induced gastroenteritis in 9 (56%) and infection in 11 (69%) of 16 genetically susceptible subjects. All infected subjects developed strong immune responses to GI.1 with a 30-fold (geometric mean titer) increase in blocking titers (BT_50_) and a 161-fold increase in GI.1-specific immunoglobulin (Ig)G titers when compared with baseline. GI.1-specific cellular responses in peripheral blood were observed 9 days postchallenge with an average of 3253 IgA and 1227 IgG antibody-secreting cells per million peripheral blood mononuclear cells.

**Conclusions:**

GI.1 Lot 001-09NV appears to be similar in virulence to previous passages of NV strain 8fIIa. The safety profile, attack rate, and duration of illness make GI.1 Lot 001-09NV a useful challenge strain for future vaccine studies aimed at establishing immune correlates.

## Teaser

A new robust platform for the purification of a novel GI.1 human norovirus stock, Lot 001-09NV, produced an infectious inoculum that can be used to compare and standardize immune response levels and efficacy of future norovirus vaccine candidates.

Diarrhea is a leading cause of morbidity and mortality in children under 5 years of age [1]. Human norovirus (HuNoV) is the leading cause of acute gastroenteritis, causing an estimated 200 000 deaths per year, mostly in young children and older adults [2]. This high disease burden is facilitated by extensive antigenic diversity between the >30 genotypes of HuNoV coupled with antigenic drift within the predominant genotype, GII.4 [3]. Antigenic drift among GII.4 strains has resulted in serial pandemic waves of disease in 2- to 7-year cycles over the past 3 decades [3, 4]. Strains outside the GII.4 genotype are associated with endemic disease and are primarily detected in young children [5]. Substantial antigenic diversity and limited cross-reactivity between genotypes have hampered the development of HuNoV vaccines. With the successful implementation of the rotavirus vaccine program in children, vaccines to HuNoV are now a high priority [6].

Most HuNoV disease is caused by strains within 2 genogroups (G), GI and GII. Genetic susceptibility to HuNoV infection is mediated by enzymes of the ABO, Lewis, and secretor histoblood group antigen (HBGA) pathways [7]. Human norovirus-HBGA interaction is genotype specific. Cross-genogroup immunity is limited among infected children [5] and vaccinated mice [8], whereas vaccination in adults, in the short-term, boosts antibodies able to broadly block binding of strains to HBGAs in a surrogate neutralization assay [9]. These blockade antibodies correlate with protection from infection and are a leading metric for vaccine immunogenicity studies [10, 11]. Other correlates of protection include mucosal immunoglobulin (Ig)A, serum IgA, and memory IgG B cells [12]. Individuals who do not have a functional secretor enzyme are genetically resistant to GI.1 infection [13, 14], yet GII.2 infection is independent of secretor type [15]. How genetic susceptibility and the immunity it shapes impacts vaccine response is not yet fully understood.

Controlled human infection models (CHIM) are being used with increasing frequency to evaluate vaccine candidates, thus methods to isolate and purify noncultivatable/recalcitrant viruses, such as HuNoV, are needed. This study was designed to develop a methodological pipeline to manufacture high-quality stocks of HuNoV for application in CHIM to elucidate the mechanisms of HuNoV susceptibility, immunity, and vaccine efficacy. After this pipeline, we prepared a stock of Norwalk virus (NV), traced its stability and sterility over a 10-year period, and demonstrated infectivity in humans of GI.1 Lot 001-09NV (NV). The development protocol yielded enough virus to generate robust immune responses in healthy adults. A similar purification approach could be applied to develop standardized, high-quality infectious virus stocks to other enteric viruses lacking high-throughput cell culture systems.

## METHODS

### Regulatory and Ethics Statement

Protocols for the source fecal sample collections and processing for challenge virus were approved by the Institutional Review Board (IRB) at the University of North Carolina Chapel Hill (no. GCRC-2355) and awarded Investigational New Drug Application (IND) 14697. The clinical trial was conducted by West Coast Clinical Trials ([WCCT] Costa Mesa, CA) under IND 18352. The clinical protocol was reviewed and approved by Aspire IRB (Santee, CA). Written informed consent was obtained from all subjects before any study related assessments. The study was registered at clinicaltrials.gov (NCT03721549).

### Preparation of Challenge Strain Virus Stocks

The challenge virus stock was prepared from a healthy donor challenged with GI.1 NV in 1999 [13] at the University of North Carolina Chapel Hill General Clinical Research Center (Supplementary Tables 1  and 2).

Thirty-two grams of GI.1 (1.8 × 10^8^ genome copies [GC]/mL) stool sample were diluted 1/5 in sterile water, vortexed for 5 minutes, centrifuged for 10 minutes at 9400 relative centrifugal force (RCF), the supernatant was removed, and the pellet was resuspended in sterile water in a washing process repeated 4 more times. The washed supernatants were pooled and centrifuged for an additional 60 minutes at 9400 RCF (Pre-Filtrate), followed by 0.22-µm filtration (Filtrate 1), and a second 0.22-µm filtration (purified bulk harvest [PBH]). Purified bulk harvest was stored at 4°C, whereas the virus titer (GC) was determined by quantitative reverse-transcription polymerase chain reaction (qRT-PCR) at Emory University [16]. The room was cleared of supplies, disinfected, and environmental monitoring repeated. A portion of the PBH was then diluted to the desired GC per mL (0.5–1.2 × 10^6^) and aliquoted into ~500 1-mL doses as the final fill and stored at less than −60°C. The remaining PBH was aliquoted and stored at less than −60°C for future packaging of sublots. Approximately, 45% (G1.1) and 72% (GII.2) of the total PBH was needed to complete the safety testing protocols described in Supplementary Table 2. The PBH and final fill were sterile, and no adventitious viruses were detected in virus-specific assays, MRC-5, VERO or HeLa cell lines, embryonic Hen eggs, or adult and newborn mice (Supplementary Table 2).

### Study Design Using the Manufactured Challenge Strain Virus Stocks

Healthy men and women between 18 and 49 years of age were recruited. A total of 40 secretor-positive, blood group O or A subjects were screened to identify 16 eligible subjects. Initially, 8 eligible subjects were assigned to the low-dose challenge (3.6 × 10^5^ GC of NV) and admitted to a private room at the inpatient challenge facility (WCCT) on the day before challenge. On the day of challenge, the 8 study subjects in this cohort were given 100 mL bicarbonate buffer (1.3% NaHCO_3_) solution to neutralize stomach acid after an overnight fast. Approximately 1 minute later, the subjects ingested the NV inoculum suspended in 100 mL distilled water.

After viral challenge, study subjects were assessed for symptoms and signs of gastroenteritis every 2 hours while awake for the first 48 hours and then every 8 hours on Day 3 through the 5-day inpatient period (+1 day if still symptomatic). All fecal and emesis samples were collected and were weighed, graded (fecal samples), and processed for NV infection using qRT-PCR [17, 18]. Saliva and blood samples were also collected at prespecified timepoints. Subjects were assessed by the nursing staff for anticipated subjective symptoms of NV illness (diarrhea, vomiting, headache, nausea, fever, abdominal cramps or pain, abdominal burgling or bloating and myalgia), as well as unsolicited adverse events (AEs).

Once all subjects in Cohort 1 (low dose) had completed their Day 14 visits, the second group of 8 subjects were challenged with a higher dose of approximately 1 × 10^6^ GC (high dose) and followed as described above.

### Illness Definitions

Acute gastroenteritis (AGE) was defined according to the criteria of Bernstein [19] as follows: (1) diarrhea (≥3 loose or liquid stools or >400 grams of loose or liquid stools produced in any 24-hour period) or (2) vomiting (≥2 vomiting episodes in any 24-hour period), or (3) 1 vomiting episode plus any loose or liquid stool in any 24-hour period, or (4) 1 vomiting episode plus at least 2 of the following 5 events: nausea, oral temperature ≥37.6°C, abdominal cramps or pain, abdominal gurgling or bloating, or myalgia in any 24-hour period during the postchallenge sequestration period. Norwalk virus infection was confirmed by fecal virus excretion detected by qRT-PCR on any day postchallenge. Norwalk virus AGE met both criteria. The modified Vesikari (17 point) scale was also used to grade the severity of illness [20].

### Secretor Assay

Saliva specimens were evaluated for H type-1 antigens secretor status by enzyme-linked immunosorbent assay (ELISA) at the Cincinnati Children’s Hospital Medical Center (CCHMC) [21, 22].

### Norwalk Virus Detection

Fecal samples were evaluated for the presence of NV by qRT-PCR assay for the viral genome at CCHMC.

### Statistical Analysis

Frequency of NV disease and infection were compared between the low- and high-dose groups by means of Fisher’s exact test. The dates of onset and resolution of AGE were determined by reviewing the subject’s clinical onset and duration of diarrheal stool (grade 3 or greater).

### Blockade Antibody Assay (BT50)

Serum samples were obtained at baseline (Day 1) prechallenge and 28 days postchallenge. In brief, biotinylated Le^b^ (Glycotech) at 2.5 µg/mL was used to coat Neutravidin-coated plates (Thermo Fisher Scientific). Two-fold dilutions of serum with GI.1 virus-like particle (VLP) were incubated at 37°C for 1 hour. The mixture of VLP and serum samples was added to HBGA-coated plates and incubated at 4°C for 2 hours. After washing, rabbit anti-GI.1 VLP polyclonal antisera (custom made by Thermo Fisher Scientific) was added and incubated at 4^o^C for 1 hour. Goat antirabbit IgG(H+L)-horseradish peroxidase (HRP) (A120-101P; Bethyl Laboratories) was used to detect bound rabbit antibody (4°C for 1 hour). With the starting dilution of 1:25, a negative value was set equal to 12.5 [11, 23].

### Peripheral Blood Mononuclear Cell Isolation and Cryopreservation

Peripheral blood mononuclear cells (PBMCs) were obtained before and at days 6 and 9 after challenge for the detection of IgA and IgG antibody-secreting cells (ASCs). Blood was collected in K_2_ EDTA Vacutainer tubes (BD), and PBMCs were isolated the same day using Lymphoprep tubes (Cosmo Bio USA, Carlsbad). The PBMCs were frozen and thawed using serum-free reagents according to the manufacturer’s instructions (Cellular Technology Ltd).

### Antibody-Secreting Cells and Enzyme-Linked Immunosorbent Assay Serum Antibodies

Antibody-secreting cells were measured using cryopreserved PBMCs and enzyme-linked immunosorbent (ELISpot) kits for IgG and IgA secreting B cells according to manufacturer’s instructions (Mabtech). Cells were cultured in triplicate with anti-IgG- or IgA-coated wells in CTL-Test Medium overnight. GI.1 VLPs made in HEK293 cells (AscentGene, Gaithersburg, MD) were biotinylated and quantitated using a biotinylation kit and BCA kit (Pierce). Spots were developed using biotinylated GI.1 VLP and streptavidin-HRP and counted at ZellNet Consulting Inc. (Fort Lee, NJ).

### Immunoglobulin G Titer Determination

Serum IgG titers were determined using a MESO QuickPlex SQ 120 instrument (Rockville, MD) following the manufacturer’s instructions and reagents. In brief, Meso Scale Discovery (MSD) Gold Streptavidin plates were blocked with 150 µl of 3% blocker B blocking solution made in phosphate-buffered tween (PBST) for 30 minutes while shaking. Plates were then washed 3 times with PBST and coated with G1.1 VLP-biotin at 0.1 µg/mL in 1% blocker B and incubated for 1 hour while shaking. Serum sample dilutions were made starting at 1:100 in 1% blocker B in PBST and diluted 1:4 down the plate. After washing, serum sample dilutions were added to the plates and incubated for 2 hours while shaking at room temperature. Plates were then washed 3 times with PBST. A total of 1 µg/mL SULFO-TAG anti-IgG antibodies, diluted in 1% blocker B, was then added to the plates and incubated for 1 hour shaking at room temperature. Plates were then washed and read after addition of 2× read buffer diluted in H_2_O.

## RESULTS

### Virus Lot Purification and Isolation

The milestones for development of the stool-derived virus stock 001-09NV is outlined in Supplementary Figure 1. A single stool sample from 1 GI.1-infected [13] donor was subjected to 5 rounds of sterile water dilution and centrifugation, and the supernatants were pooled (Pre-Filtrate) before being sterile filtered (Filtrate 1) twice to generate the PBH. Aliquots before the filtration step contained bacteria and fungi. The postfiltration samples were free of bacteria and fungi and all other tested pathogens (Supplementary Table 2). A portion of the PBH was further diluted and vialed in 1-mL doses, recorded as Lot 001-09NV (GI.1). The remainder of the PBH was stored in bulk for future sublots. As expected, the stool-derived product contained endotoxin, although at levels 3- to 8-fold less than GI.1 8FIIb, a HuNoV strain used in previous CHIM [13] (Supplementary Table 2). The virus stock was stable, sterile, and infectious in storage as tested over the past 10 years (Table 1). A request for use of 001-09NV virus stock in CHIM was submitted and approved to the US Food and Drug Administration (FDA), under an IND (14697).

**Table 1.** Virus Stock Stability and Sterility Over Time^a^

	001-09NV		
Year	GC/mL	Sterility	Endotoxin EU/mL
2009 Manufacture	5 × 10^5^	Negative for growth	235
2011 IND	6 × 10^5^	Negative for growth	N/D
2018 001-09NV Trial	3.6 × 10^5^	Negative for growth	165
2019 001-09NV Trial	1.4 × 10^5^	Negative for growth	155

Abbreviations: EU, endotoxin units; GC, genome copies; IND, Investigational New Drug; N/D, not determined.

^a^Human noroviruses final fill lots were tested for virus stability (mean GC per mL), sterility, and endotoxin at manufacture, US Food and Drug Administration IND Application submission, initiation of studies, and annually during ongoing studies. Testing was not performed during years of nonhuman use.

### Clinical Outcome

All subjects enrolled in the challenge study were healthy men and women 18–49 years old that were secretor-positive and blood group O or A. The mean age of the subjects was 33 years old in the low-dose group and 40 years old in the high-dose group (Table 2). No unexpected/unsolicited AEs or serious AEs were reported. Unexpected/unsolicited AEs exclude gastroenteritis symptoms because gastroenteritis is expected after ingestion of live norovirus. Expected/solicited AEs included nausea, diarrhea, vomiting, abdominal pain, fever, muscle aches, headache, and chills. In previous studies, norovirus challenge strains have been safely administered [19, 20, 24].

**Table 2.** Demographic Characteristics by Dose Cohort

Baseline demographics	Dose Cohort^a^		
	Low	High	All
	n = 8	n = 8	n = 16
Mean age (years)	32.5	39.9	36.3
Gender			
Female	3 (37)	4 (50)	7 (44)
Male	5 (63)	4 (50)	9 (56)
Ethnicity			
Hispanic	2 (25)	4 (50)	6 (38)
Not Hispanic	6 (75)	4 (50)	10 (63)
Race			
White	4 (50)	5 (63)	9 (56)
Black	4 (50)	2 (25)	6 (38)
Asian	0	1 (13)	1 (6)
Body mass index (mean)	25.7	27.1	26.4
Blood Type			
A	3 (37)	5 (63)	8 (50)
O	5 (63)	3 (37)	8 (50)
Secretor status positive	8 (100)	8 (100)	16 (100)

In the low-dose group, 5 of 8 subjects (63%) met the criteria for AGE and were also positive for NV by qRT-PCR (Table 3). The other 3 subjects in the cohort were well and NV negative. In the high-dose group, 6 of 8 (75%) were NV positive; 4 met the AGE criteria, and 2 others had milder diarrhea that did not meet the definition of AGE. One subject was well and NV negative, and another subject had diarrhea and was negative for both NV GI.1, GII.4, and other enteric pathogens (data not shown). Overall, there were no statistical differences in outcome among treatment groups. Among those subjects who had AGE and were NV positive, the illness appeared more severe in the high-dose group as measured by diarrheal stool output and vomiting. The onset of illness was shorter, and the duration of illness was longer in the high-dose group. The Vesikari score was also higher in the high-dose group compared with the low-dose group (6.3 vs 4.8), and the mean maximum body temperature was also slightly higher in the high-dose group (Table 4). None of the differences between dose groups was statistically significant. The symptoms recorded among the 9 subjects with AGE and who were NV positive were in general mild to moderate in nature (data not shown).

**Table 3.** Summary of the Correlation Between Acute Gastroenteritis and NV Positivity by Cohort

Category		Dose Cohort		
		Low	High	All
Illness	Infection	n = 8	n = 8	n = 16
AGE	NV^+^	5 (62.5)	4 (50)	9 (56.3)
Well	NV^+^	0	2 (25)	2 (12.5)
AGE	NV^−^	0	1 (12.5)	1 (6.3)
Well	NV^−^	3 (37.5)	1 (12.5)	4 (25)

Abbreviations: AGE, acute gastroenteritis; NV, Norwalk virus.

**Table 4.** Characterization of Diarrhea in Subjects With Acute Gastroenteritis and NV Positivity by Cohort (by Mean)

Characteristic	Low Dose	High Dose	All Subjects
Number of subjects	5	4	9
No. diarrheal stools	3.4	8.3	5.6
Total wt. (g)	599	880	723.7
No. vomiting episodes	1.5	2.7	2.2
Highest temperature (°C)	37.3	37.5	37.4
Time to onset (h)	52.5	36.9	45.6
Duration diarrhea (h)	10	20.7	14.7
Vesikari score	4.8	6.3	5.4

Abbreviations: h, hours; NV, Norwalk virus; wt, weight.

Norwalk virus shedding was highly correlated with diarrheal stool (grade 3 or greater). In the low-dose group, shedding ranged from 10^5^ to 10^10^ GC/gram of stool. Virus was also detected in emesis in 1 of 2 subjects. Similar results were observed in the high-dose group. The level of viral shedding did not correlate with the amount of diarrheal stool. Norwalk virus was detected in 3 of 16 subjects on day 9.

### Immunological Responses

Serological and cellular assays were used to evaluate the immunological responses to norovirus challenge. Subjects that were positive for NV shedding were further subclassified into positive or negative for AGE (Table 5), and their immune responses were compared.

**Table 5.** Cellular and Serological Immune Responses Analyzed by Clinical Outcome

	All NV^+^	NV^+^/AGE	NV^+^/Well	NV Negative^a^
No. of Subjects	n = 11	n = 9	n = 2	n = 5
BT_50_ (GMT)				
Day 1	39	35	59	100
Day 28	1205	1048	2263	130
GM fold increase	31	30	38	1.3
≥4-fold increase	11	9	2	0
Serum IgG (GMT)				
Day 1	3200	2743	6400	18 102
Day 28	436 241	442 392	409 600	18 102
GM fold increase	136	161	64	0.9
≥4-fold increase	11	9	2	0
IgA ASC (Avg)				
Day 1	1	1	1	1
Day 6	4	4	2	3
Day 9	3011	3253	1920	6
IgG ASC (Avg)				
Day 1	0	0	0	0
Day 6	1	2	0	1
Day 9	1288	1227	1564	2

Abbreviations: ASC, antibody-secreting cells; Avg, average; GM, geometric mean; GMT, GM titer; Ig, immunoglobulin; NV, Norwalk virus;

^a^One subject did not return on day 28 and was excluded from BT50 and IgG enzyme-linked immunosorbent assay day 1 baseline analysis.

#### BT_50_ Blockade Assays

Blockade assays were used as the primary endpoint to test for subject seroconversion after viral challenge. Subjects with a geometric mean fold rise (GMFR) >4 from baseline titer were considered successfully seroconverted for blockade antibody. On day 28, all subjects that were positive for norovirus had an average BT_50_ titer of 1205, which represented a GMFR >30-fold from day 1 with a 100% seroconversion rate (11 of 11). It is remarkable that, within the AGE group, 4 of 16 subjects (25%) and 2 of 16 subjects (12.5%) had a 32- and a 64-fold increase in BT_50_ titer by day 28, respectively. In contrast, none of the subjects negative for NV seroconverted for blockade antibody by day 28, only showing a 1.3-fold BT_50_ titer increase from day 1.

#### Serum Immunoglobulin G Titer Determination

GI.1-specific IgG responses closely matched the trends observed in BT_50_ assays. Norwalk virus-positive subjects, with a range of titers between 204 800 and 3 276 800 on day 28, had an average 136-fold GMFR from baseline in ELISA titers, as measured by MSD. Within the AGE group, 4 of 16 subjects (25%) and 1 of 16 subjects (6.25%) had a 256-fold and a 1024-fold increase in serum IgG titer by day 28, respectively. Only 1 subject that did not meet the criteria to be considered positive for AGE responded with a 64-fold increase in ELISA titers by day 28. However, none of the subjects who were negative for AGE seroconverted. As expected, there was a high positive correlation between serum BT_50_ and NV-VLP IgG ELISA titers both at baseline (Pearson r = 0.91) and at day 28 (Pearson r = 0.88).

#### Antibody-Secreting Cell Assays

Antibody-secreting cell responses (IgA and IgG) were measured in all subjects at 3 different timepoints: day 1 (prechallenge), day 6 and day 9 (5 and 8 days after challenge, respectively). Counts were low/negligible on days 1 and 6 but rose to high ASC numbers on day 9 in NV-positive subjects (Table 5). On average, NV-positive subjects with AGE had 3253 IgA ASC and 1227 IgG ASC spot counts per 1 × 10^6^ PBMCs on day 9, showing a bias toward IgA versus IgG. It is remarkable that 8 of 16 subjects (50%) had IgA ASC spot counts ≥2000 on day 9. Immunoglobulin A ASC counts were approximately 50% higher among subjects who were ill compared with the 2 subjects who were asymptomatic (Table 5). As expected, subjects that were negative for NV had negligible ASC spot counts on any day.

All 11 NV-positive subjects showed strong serum and ASC responses, whereas the 5 subjects who were NV negative had no detectable immune responses (Table 5). Three of 16 subjects (19%) had baseline BT_50_ GI.1 titers of ≥1:200 and showed no NV-related symptoms, supporting previous observations indicating that blockade antibody titer correlates with protection from symptomatic norovirus infection [10, 11]. Two other NV-negative, asymptomatic subjects had BT_50_ titers of 25 and 50, respectively, suggesting additional, as yet undefined, mechanisms of norovirus illness protection.

## DISCUSSION

Vaccine efficacy studies using a human challenge design require large quantities of well characterized virus stocks that meet FDA sterility and purity requirements for use in humans. Using a sample collected during our previous HuNoV infectivity study, we prepared virus stock for the genogroup I protype strain, GI.1 NV [13]. These strains are good HuNoV vaccine testing candidates because they circulate in the population and the environment but are typically not associated with pandemic levels of disease [3, 18]. Unlike the pandemic GII.4 HuNoV strains [25], the results of challenge studies with GI.1 and GII.2 are less likely to be complicated by high levels of either community-acquired infections or herd immunity within the test population, while providing key information on strain susceptibility and vaccine-induced breadth of immunity.

This study describes the infectivity of the GI.1 NV Lot 001-09NV in humans, initially developed as a tool to better characterize and standardize immune responses generated by CHIM infections. The data presented here support that 001-09NV infectivity has been maintained over time after extensive passaging in healthy human volunteers and, more importantly, is similar to that of its parent strain, 8FIIa, previously tested in several trials [13, 26]. In the current study, all subjects that were positive for stool NV had a strong serological and cellular immune response to NV. Specifically, substantial increases in serum antibody responses were observed, with a GMFR from baseline of 31 in BT_50_ and 136 in IgG titers measured on day 28 postchallenge. Previous studies with Vaxart’s oral norovirus vaccine have shown that such immunization elicited a cellular response that was skewed toward IgA with an average IgA ASC spot count of 561 per 1 × 10^6^ PBMCs (0–1600) among subjects who received the highest dose at day 7 postimmunization [23]. In the present study, challenged subjects also showed a stronger cellular IgA response, although with a higher variability in magnitude, and an average IgA ASC spot count of over 2000 (range, 0 to 6800) on day 9 postchallenge, suggesting that responses to NV infection are considerably higher than those elicited by vaccination. For varicella-zoster virus (VZV), the antibody responses after immunization with the VZV vaccine (live-attenuated), while still protective, have been estimated >10-fold lower than those elicited after natural infection [27]. In our study, all 11 subjects that were infected with NV and ill had a 4-fold increase in BT_50_ titers from baseline by day 28, consistent with previous challenge studies with other GI.1 inoculums [11]. The magnitude of the BT_50_ titers on day 28 was similar to that reported in some previous challenge studies [11, 28]. Overall, our data show that the seroresponses observed after NV challenge are comparable to those observed in previous controlled human infection studies with similar strains of genogroup GI.1 [20].

The present study provides a robust platform and a pipeline for studies with other noncultivable enteric viruses of importance to human and veterinary health such as sapovirus, reovirus, or adenovirus. Our approach furthers other vaccine studies because it opens the possibility to use Lot 001-09NV for studying protective immunity markers against norovirus infections. Virus neutralization assays are not available for NV. Historically, BT_50_ titers have been regarded as one such protective immunity marker. In this study, 3 subjects had baseline BT_50_ titers of ≥200 and did not develop NV-related gastroenteritis, although one of them had an increase in antibody responses postchallenge. Elevated baseline titers were also associated with a lower risk of illness in a previous challenge study, particularly in placebo subjects [11, 29].

## CONCLUSIONS

In summary, we have found that Lot 001-09NV is similar in virulence to previous passages of NV strain 8FIIa, with a similar safety profile, time to disease onset, and duration of illness in challenged subjects. All of these attributes make this lot a very useful challenge isolate to compare and standardize immune response levels and efficacy of various vaccine candidates. The large number of high virus titer stool samples collected here could serve as valuable sources of virus to pilot development of the human intestinal enteroid in vitro cell culture system, which allows for NV replication in an isolate-specific manner [30, 31].

## Supplementary Data

Supplementary materials are available at *The Journal of Infectious Diseases* online. Consisting of data provided by the authors to benefit the reader, the posted materials are not copyedited and are the sole responsibility of the authors, so questions or comments should be addressed to the corresponding author.

jiz540_suppl_Supplementary_Figure_1

jiz540_suppl_Supplementary_Table_1

jiz540_suppl_Supplementary_Table_2

## Notes

***Acknowledgments.*** We thank Patty Orozco-Cronin (Vaxart); the clinical staff at West Coast Clinical Trials (WCCT); Monica McNeal, Weiming Zhong, and Xi Jason Jiang (Cincinnati Children’s Hospital Medical Center); and Christine L. Moe, Marina Fernandez, and Pengbo Liu (Emory University).

Author contributions. Challenge virus and Investigational New Drug were developed jointly between the University of North Carolina (UNC) and Emory University (R. S. B., L. C. L., J. S. L., and A. C. S.) with D. J. W. providing medical oversight in donor screening and selection. S. J. G. and K. G. managed operational activities with WCCT, who conducted the clinical trial with financial support from Vaxart. K. L. and S. S. performed the immunological assays at Vaxart. R. M. and S. N. T. analyzed the immune response after challenge. R. M., L. C. L., R. S. B., S. J. G., and D. N. T. wrote the manuscript with input from all authors.

***Financial support.*** This work was funded by Vaxart Biosciences, Inc., and grants from the National Institutes of Health (56AI106006, U19 AI109761; Centers of Excellence for Translational Research AI056351 [to R. S. B.]; AI23946, RR00046, and GM63228 [UNC General Clinical Research Center]), the Wellcome Trust (203268/Z/16/Z; to R. S. B.), and the US Department of Agriculture-National Institute of Food and Agriculture (2018-07410; to J. S. L.).

***Potential conflicts of interest.*** Vaxart purchased the challenge virus from UNC. R. S. B., D. J. W., L. C. L., and A. C. S. are employees of UNC. Vaxart analyzed the immune responses after the WCCT challenge study. R. M., S. J. G., K. G., K. L., S. S., S. T., and D. N. T. are employees of Vaxart. All authors have submitted the ICMJE Form for Disclosure of Potential Conflicts of Interest. Conflicts that the editors consider relevant to the content of the manuscript have been disclosed.
