# Supplementary material for: Production and Clinical Evaluation of Norwalk GI.1 Virus Lot 001-09NV in Norovirus Vaccine Development
Source: J Infect Dis. 2019 Oct 20;221(6):919–26. doi: 10.1093/infdis/jiz540 (PMC7050988; doi:10.1093/infdis/jiz540)

**Supplementary Figure S1.** Milestones in developing human norovirus challenge virus stocks including key intermediates (orange) and production steps (blue) in the manufacturing process.

Donors from the original GI.1 virus challenge (1999-2001) were screened and selected based on positive infection status and the presence of at least 3 infected stools stored at -80°C [13, 15]. Under the approved IRB protocol, donor’s contact information was identified from clinical records, information from UNC-GCRC staff, and as a last-case, from a commercial service. Donors who were selected and had valid contact information were re-contacted by study staff to explain the study and a copy of the informed consent was shared with them. Two donors provided informed consent for further pathogen testing of archived samples, a current health evaluation including pathogen testing and approval for use of existing samples. At the time of original virus challenge, donors were in good health as determined by health screenings at University of North Carolina Hospitals. An additional health screening was completed between 2005 and 2006 when the donors were recalled for participation in the stock virus safety testing screening. At recall, donors were negative for HIV and hepatitis viruses, tuberculosis, and other tested pathogens (**Supplementary Table 1**). Additionally, in 2009, at the time of virus stock manufacture, archived stool and serum collected from the donors at the time of original virus infection were tested for HIV and hepatitis viruses by WuXi-Apptec (protocols C30635.04, C30665.04, C30703.04, C30730.05, C32827) (**Supplementary Table 1**). As per IND 14697, for virus stock preparation, an isolation laboratory at the Department of Epidemiology, University of North Carolina Chapel Hill with controlled access and environmental microbial monitoring was established. Staff were trained in production standard operating procedures (SOPs) and supplies and reagents were sequestered for the processing. Only one virus stock was processed at a time. Before processing, the room was cleaned floor to ceiling with an EPA approved hospital surface disinfectant. Environmental contaminants were assessed by surface and air monitoring before and after each production run as well as before the final dilution and dispersion of the final virus stock into doses. All measurements were within acceptable limits, as per IND 14697.

SN, supernatant; FDA, Food and Drug Administration; IND, Investigational New Drug Application.


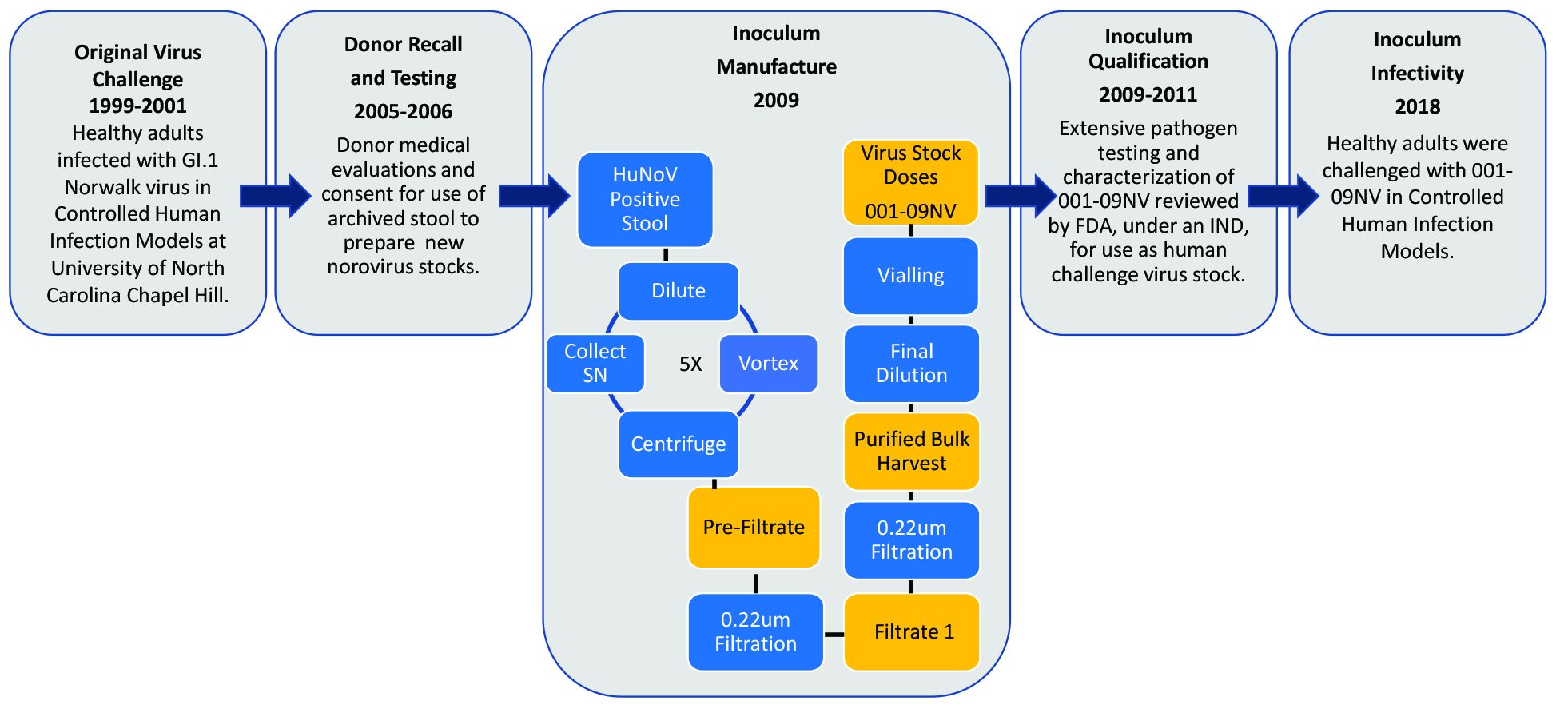

Supplement: jiz540_suppl_Supplementary_Figure_1 [file jiz540_suppl_supplementary_figure_1.docx]
