# Supplementary material for: Production and Clinical Evaluation of Norwalk GI.1 Virus Lot 001-09NV in Norovirus Vaccine Development
Source: J Infect Dis. 2019 Oct 20;221(6):919–26. doi: 10.1093/infdis/jiz540 (PMC7050988; doi:10.1093/infdis/jiz540)
Supplement: jiz540_suppl_Supplementary_Table_1 [file jiz540_suppl_supplementary_table_1.docx]

**Supplementary Table 1.** Donor samples were negative for non-human norovirus tested pathogens.

|  | Recall Screening | Archived Serum | Archived Stool |
| --- | --- | --- | --- |
|  | **2005-2006^a^** | **2009^b^** | **2009^b^** |
| HIV 1 | Negative | Negative | Negative |
| HIV 2 | Negative | Negative | Negative |
| Hepatitis A | Negative | Negative | Negative |
| Hepatitis B | Negative | Negative | Negative |
| Hepatitis C | Negative | N/D | Negative |
| Hepatitis E | Negative | N/D | N/D |
| HTLV | Negative | N/D | N/D |
| Syphilis | Negative | N/D | N/D |
| Tuberculosis | Negative | N/D | N/D |

**^a^** Donors were screened for markers of infection in serum or stool when recalled for a health evaluation in 2005-2006.

^b^ Serum and stool archived in 1999/2001 during original virus infection were tested in 2009 for signs of infection.

ND, not determined.
