# Supplementary material for: Production and Clinical Evaluation of Norwalk GI.1 Virus Lot 001-09NV in Norovirus Vaccine Development
Source: J Infect Dis. 2019 Oct 20;221(6):919–26. doi: 10.1093/infdis/jiz540 (PMC7050988; doi:10.1093/infdis/jiz540)
Supplement: jiz540_suppl_Supplementary_Table_2 [file jiz540_suppl_supplementary_table_2.docx]

**Supplementary Table 2.** Adventitious agent and endotoxin testing of virus stock manufacturing intermediates and products.

| Sample | Test | Test Protocol | Specification | 001-09NV | 001-095M |
| --- | --- | --- | --- | --- | --- |
| Pre-Filtrate | Bioburden | WuXi–Apptec 30751 | Record Result | Aerobes 1, Fungi <1 | Aerobes TNTC^a^, Fungi 3 |
| Filtrate 1 | Sterility | WuXi–Apptec 30744 | Negative for growth | Negative for growth | Negative for growth |
| Purified Bulk | Sterility | WuXi–Apptec 30744 | Negative for growth | Negative for growth | Negative for growth |
| Harvest | Sterility B/F^b^ | WuXi–Apptec 30736 | Pass | Pass | Pass |
|  | Mycoplasma | WuXi–Apptec 30200 | Negative | Negative | Negative |
|  | In vitro Virus | WuXi–Apptec 3700D | Negative | Negative | Negative |
|  | In vivo Virus | WuXi–Apptec 30027 | Negative | Negative | Negative |
|  | Astrovirus | CDC RT-PCR | Negative | Negative | Negative |
|  | Adenovirus | CDC RT-PCR | Negative | Negative | Negative |
|  | Sapovirus | CDC RT-PCR | Negative | Negative | Negative |
|  | GIV Norovirus | CDC RT-PCR | Negative | Negative | Negative |
|  | Rotavirus | CDC RT-PCR | Negative | Negative | Negative |
|  | Endotoxin | WuXi–Apptec 30739 | < 500,000 EU/ml | 3640 EU/ml | 105,000 EU/ml |
| Final Fill Dose | Sterility | WuXi–Apptec 30744 | Negative for growth | Negative for growth | Negative for growth |
|  | Sterility B/F | WuXi–Apptec 30736 | Pass | Pass | Pass |
|  | Mycoplasma | WuXi–Apptec 30200 | Negative | Negative | Negative |
|  | Endotoxin | WuXi–Apptec 30739 | < 5000 EU/ml | 235 EU/ml | 1310 EU/ml |

Products post-filtration are sterile and free of any detected adventitious viruses. Endotoxin levels are below levels in a HuNoV stock previously used in CHIMs (8fIIb, 3770 EU/ml, determined as above).

^a^ Too numerous to count

^b^ Neither encouraged or discouraged bacterial or fungal growth.
